# e-Learning in Phoniatrics and Speech-Language Pathology: Exploratory Analysis of Free Access Tools in Augmentative and Alternative Communication

**DOI:** 10.2196/63392

**Published:** 2025-06-26

**Authors:** Jessica Büchs, Christiane Neuschaefer-Rube

**Affiliations:** 1 Clinic for Phoniatrics, Pedaudiology & Communication Disorders, University Hospital and Medical Faculty, RWTH Aachen University, Pauwelsstraße 30, Aachen, 52074, Germany, 49 2418038717

**Keywords:** E-learning, digital learning, augmentative and alternative communication, speech-language pathology, phoniatrics, communication disorders, complex communication needs, communication aid

## Abstract

**Background:**

Augmentative and alternative communication (AAC) is a therapeutic approach and modality of expression for patients with limited or no expressive language. Speech-language pathologists and phoniatricians need to be competent in AAC to treat patients with complex communication needs. For knowledge acquisition and enhancement in AAC, a significant number of e-learning tools are available. To improve e-learning in AAC, it is essential to understand the attributes of these tools, such as formats, content areas, learning styles, or learning goals. However, these structures have yet to be investigated.

**Objective:**

With this study, we aimed to (1) explore free access AAC e-learning tools that are appropriate for students and professionals of phoniatrics and speech-language pathology; (2) gain insight into formats, content areas, learning styles, and learning goals; and (3) investigate structural differences within and between basic and advanced learner level.

**Methods:**

In 2023, we conducted a systematic web-based search with defined search terms in PubMed, peDOCS, Google Scholar, Google, the Apple App Store, and the Google Play Store in accordance with the PRISMA (Preferred Reporting Items for Systematic Reviews and Meta-Analyses) 2020 guidelines and piloting a protocol for data abstraction and validation. Inclusion criteria were free access, a mandatory minimum AAC content, and the use of the English or the German language. Social networks, video-sharing platforms, blogs, and forums were excluded. We analyzed formats (websites, online courses, apps, and podcasts), content areas (types of AAC, diagnostics, therapy, and other content areas), learning styles (visual, auditory, and audio-visual), and learning goals (receptive and performative) within and between basic and advanced level tools.

**Results:**

We identified 131 tools, of which 57 (43.5%) were basic level and 74 (56.5%) were advanced level. Of these 131 tools, 105 (80.2%) were websites, 21 (16%) were online courses, 3 (2.3%) were apps and 2 (1.5%) were podcasts. Only 12 out of 74 (16.2%) tools for advanced learners offered performative tasks. For basic learners no such tasks could be identified. For learning style, all basic tools and most of the advanced level tools were “visual (text)” (57/57, 100% basic vs 66/74, 89.2% advanced). In terms of content, advanced level tools pertained more often to “diagnostics” (28/57, 49.1% basic vs 65/74, 87.8% advanced) and “therapy” (17/57, 29.8% basic vs 64/74, 86.5% advanced). Advanced level courses were more likely online courses (2/57, 3.5% basic vs 19/74, 25.7% advanced) and more often showed audio-visual learning styles compared with basic level tools (5/57, 8.8% basic vs 27/74, 36.5% advanced).

**Conclusions:**

Our study showed that free-access AAC tools for phoniatrics and speech-language pathology varied in formats, content areas, learning styles, and learning goals. Furthermore, we found differences within and between learner levels. Thus, we established a basis for future research in e-learning in AAC.

## Introduction

### Background

Augmentative and alternative communication (AAC) describes ways to support or replace spoken words for people who are unable to speak or communicate effectively using natural speech. Types of AAC include facial expressions, gestures, signs, cards with symbols, letterboards, or the use of electronic communication aids such as voice output devices [1-3]. Some published works concur that sign languages belong to AAC [4,5] while others state that they are not considered AAC [6]. The types of AAC are categorized into “unaided” and “aided,” depending on whether the patient uses solely their body to communicate or a communication aid [7]. Therefore, patients with limited expressive language may use various types of AAC to communicate.

A significant number of patients may benefit from AAC. Numerous medical conditions are known to be the cause of severe speech and language impairments that require AAC. These medical conditions are genetic disorders (eg, Down syndrome [8,9], Rett syndrome [10], and Angelman syndrome [11]), neurological impairments (eg, cerebral palsy [7,12-14], aphasia [7,15,16], dysarthria [7], apraxia of speech [7], Parkinson's disease [14]), motor neuron diseases (eg, amyotrophic lateral sclerosis and progressive muscular atrophy) [14,17,18], intellectual and developmental disabilities [19], severe hearing loss or deafness [20,21], different states of consciousness [22-24], postsurgical states affecting speech (eg, laryngectomy and tracheostomy) [7,25], and other medical conditions such as dementia [14], multiple sclerosis [14], autism spectrum disorder [7,14,26,27], visual impairment [28-30], locked-in-syndrome [31], and treatment in intensive care [32,33]. Creer et al [14] estimate that approximately 0.5% of the population of the United Kingdom are potential AAC users. Thus, AAC is a common therapeutic approach for various kinds of patients.

Patients with special communication needs, who may benefit from AAC, seek treatment in hospitals and established practices for phoniatrics and speech-language therapy [4,34]. Zinkevich et al [35] demonstrated the importance of the implementation of AAC in medical service delivery. Accordingly, phoniatricians and speech-language pathologists need to be competent in AAC. In preparation of this study, we formulated 3 main competences that phoniatricians and speech-language therapists may need in order to provide service to potential AAC users. First, phoniatricians and speech-language therapists should be able to identify patients that benefit from AAC. Second, they should be able to differentiate the types of AAC. Third, they should be able to decide which patients may benefit from what types of AAC. Thus, clinical professionals in phoniatrics and speech-language pathology (SLP) require specific knowledge in AAC to treat patients who may benefit from AAC.

What are sources of knowledge when it comes to AAC? Medical students are not obligatorily taught AAC at university. In Germany, even university students of SLP may have limited knowledge of AAC. In view of this fact, students as well as clinical professionals may search the web for information on AAC. Phoniatricians and speech-language pathologists use e-learning tools to acquire and enhance their expertise in their respective field [36,37], among other learning methods. Consequently, AAC e-learning tools are relevant in phoniatrics and SLP.

e-Learning tools can be described by their structures including formats, content areas, presentation modes, sensory modes, learning goals, target groups, and other describing structures [38]. In terms of formats, a tool can be a website, an online course, an app, a podcast, or another digital format. The learning styles of e-learning tools can be visual, auditory, or audio-visual [39,40]. Furthermore, e-learning tools have either a receptive or a performative learning goal [40]. In addition, e-learning tools may vary in content. Consequently, there are various possibilities to classify e-learning tools. This study focusses on the following e-learning structures: formats, content areas, learning styles, and learning goals.

Why is it essential to gain insight into the nature of AAC e-learning tools? Certain attributes such as “online course,” “audio-visual,” or “performative” characterize e-learning tools. This characterization may attract a specific group of learners (eg, learners who prefer a visual learning style may use websites with diagrams). A quantitative analysis could identify predominant or lacking structures. A “baseline” or status quo of the e-learning structures of AAC tools would be a starting point for further investigations, understanding and improving e-learning in AAC in the long term. However, the nature of AAC e-learning tools has yet to be investigated.

### Goals of This Study

With this study, we aim to (1) explore free access AAC tools that are appropriate for e-learning in phoniatrics and SLP, (2) gain insight into the e-learning features of these tools (formats, content areas, learning styles, and learning goals), and (3) investigate structural differences within and between basic and advanced level tools. Furthermore, our goal is to establish a basis for future research in which we plan to test and evaluate a newly developed AAC e-learning tool for students of medicine and SLP.

### Previous Work and Contribution

The study of Lin and Neuschaefer-Rube [38] in 2021 was about the onset of e-learning studies that discussed the improvement of e-learning in SLP, phoniatrics, and otolaryngology. They investigated the e-learning structures of tools in SLP, phoniatrics, and otolaryngology. Differences within and between academic-level learners and clinical-professional learners were found in terms of formats, content areas, and learning goals. Thus, their study presented an initial overview of existing e-learning tools in the interdisciplinary field of SLP and phoniatrics.

Our study contributes to the improvement of e-learning in AAC as being one of the many fields of interest in phoniatrics and SLP. By systematically searching the web for AAC e-learning tools, we gain an understanding of the overall quantity of AAC tools and their availability. An analysis of the e-learning tools in AAC provides further insight into the formats, content areas, learning styles, and learning goals that were state of the art during the time of search. Accordingly, this study should add new findings to the ongoing e-learning research in phoniatrics and SLP.

## Methods

### Protocol, Checklist, and Registration

Our study is an original, new, and exploratory investigation within the interdisciplinary field of medicine and SLP that targeted interactive learning tools on a niche topic. Therefore, our study does not conform to the conventional framework of a systematic review. To find the tools, a novel approach was necessary. To adhere the tenets of good scientific practice, we developed structured protocols for the following processes: systematic web-based search, tool selection, and data abstraction and validation. For transparent, complete, and accurate reporting, we proceeded in accordance with PRISMA (Preferred Reporting Items for Systematic Reviews and Meta-Analyses) 2020 [41]. The checklist is provided in Multimedia Appendix 1. This study was registered at our institution Rheinisch-Westfälische Technische Hochschule (RWTH) Aachen University (no CTC-A 24‐036) and has not been registered elsewhere.

### Systematic Web-Based Search

In the summer of 2023, we conducted a systematic web-based search in “Google” (Google Search), Google Scholar, PubMed, peDOCS, the Apple App Store, and the Google Play Store using company owned devices. The use of nontraditional information sources such as “Google” and Google Scholar was necessary in the search for interactive tools. We combined general, academic, medical, and educational search engines to obtain optimal results. The combination of medical and educational databases was chosen since AAC is an interdisciplinary field. By searching App store, we targeted apps that teach about AAC. Gray literature search was not conducted. We used various search terms in English (American and British) and German ranging from specific to broad in the fields of education and medicine. Our IT center ensured that the IP address would not affect the search results. The same computer was used for all web-based searches. However, random checks with a different computer were done to ensure the results were the same. Multimedia Appendix 2 shows a protocol of our search including dates of search, search engines, search terms, number of records screened, and the final tools that met the inclusion and exclusion criteria.

### Tool Selection Process

#### Records Identified, Removed, and Screened for Eligibility

Thousands of search results had been obtained, thus necessitating the implementation of limits for the screening process. We set a limit of the first 25 search results for each broad search term and a limit of the first 10 search results for specific terms. If the number of search results was less than 25 or 10, respectively, the records were limited to that number. In total, this strategy led to 1616 search results that were screened for eligibility. The screening was done by author [JB]. Automation tools were not used. We estimate that half of the records were discarded because they had no relation to AAC. The rationale behind this can be attributed to the polysemy of the acronym “AAC.” For that matter, the originally planned search terms with the German abbreviation “UK” were dismissed. In addition, all tools pertaining to how AAC users can learn digitally, rather than how one can learn about AAC digitally, were removed, as well as duplicates.

#### Screening for Inclusion and Exclusion Criteria

After the initial filtering, approximately 700‐800 tools remained to be screened for inclusion and exclusion criteria. Those results underwent a selection process based on access, content, and language. According to access, only tools that had a free, immediate, and full access were chosen for this study. Registration and email confirmation were tolerated. In terms of content, we defined a mandatory minimum. To be selected for this study, a tool had to contain a definition of AAC. In addition, a tool had to at least cover one of the following content areas: types of AAC, diagnostics, or therapy to ensure the tools were appropriate for the fields of phoniatrics and SLP. Tools, in this case apps, that only taught sign languages or functioned solely as a talker were excluded. Regarding language, only tools in English and German were included. However, social networks, video-sharing platforms, blogs, and forums were excluded. The search results obtained from the academic search engines yielded research papers. Since we targeted interactive tools rather than books and papers, we screened the results for links to free tools (eg, online courses). Reference lists were not reviewed. Using this technique, we found 3 websites that we added to our tool list. Nevertheless, these 3 tools had also previously been identified in the Google search. The tool list was supplemented by 2 websites added by the authors. Again, duplicates were removed. Finally, 131 tools were left for data abstraction.

### Data Abstraction and Validation Process

To ensure a clear and concise method for data abstraction, we piloted a protocol (Multimedia Appendix 3). The data abstraction was done by author JB. Automation tools were not used. Author CNR checked for validity. Both authors followed the protocol and reported no bias. Uncertainties were solved in an interdisciplinary discussion between JB as a speech-language pathologist and CNR as a medical professor.

### Tool Analysis

The tools were analyzed by basic and advanced level in terms of the following e-learning structures: formats (websites, online courses, apps, and podcasts), content areas (types of AAC, diagnostics, therapy, and other content areas), learning styles (visual, auditory, and audio-visual), and learning goals (receptive and performative).

#### Learner Level

We defined the criteria for basic-level and advanced-level tools based on our clinical and teaching experience. Basic level tools provided only general information. This level is appropriate for learners with no previous knowledge of AAC such as students of medicine and SLP. Advanced level tools exceeded general information and required either previous knowledge of AAC or clinical experience. Advanced-level tools are appropriate for students of medicine and SLP with previous knowledge as well as for professionals with clinical experience in AAC.

#### Formats

The tools of this study were either websites, online courses, apps, or podcasts. We analyzed websites of speech-language pathologists, clinics, consultation offices, self-help groups, institutions for special needs, and specific websites such as the website of the American Speech and Hearing Association and the German Society of AAC. We participated in online courses from universities and other teaching institutions. The online courses were recorded lectures, presentations, or modules on learning platforms. With regards to apps, we targeted those that taught AAC. However, almost all apps functioned as a talker or trained the user in sign language while lacking a definition of AAC. Consequently, these apps were excluded from this study, leaving only 3 apps to our analysis. Finally, 2 podcasts were analyzed, although we had not explicitly searched for podcasts. In conclusion, the 4 formats in this study were websites, online courses, apps, and podcasts.

#### Content Areas

We analyzed the tools’ content according to our previously defined 3 main competences that professionals in SLP and phoniatrics need: (1) knowledge about the types of AAC, (2) identification of potential AAC users, and (3) assignment of a type of AAC to a patient. Accordingly, the following 4 content areas were defined. The first content area was “types of AAC” for tools that provided a detailed explanation of at least 1 type of AAC or an overview of the types of AAC. The second content area was “diagnostics” for tools that identified at least 1 medical condition of AAC users. The third content area was “therapy” for tools that provided at least 1 example of a patient and their type of AAC. The fourth content area was “other content areas” for tools that provided other valuable information. This information could be downloads (eg, communication boards, sign language cards, collections of symbols, and other material), glossaries or descriptions of specific approaches in AAC. Thus, the 4 content areas in this study were “types of AAC,” “diagnostics” “therapy,” and “other content areas” to ensure the tools meet the needs of learners in phoniatrics and SLP.

#### Learning Styles

A total of 4 learning styles were identified, depending on whether the tools contained texts, pictures, diagrams, audio-files, or videos. When information was received via vision (eg, reading a text, interpreting diagrams, and looking at pictures), the tools were “visual (text)” or “visual (picture or diagram).” Auditory tools were audio-files where information was received via hearing. The audio-visual learning style was assigned to videos.

#### Learning Goals

Inspired by Lin and Neuschaefer-Rube [38], we defined the following learning goals for this study: “receptive” and “performative.” A tool was “receptive” when information was only transmitted via reading or listening (ie, passive consumption). When a tool required action, it was “performative.” Performative tools were further differentiated into “directive” and “guided discovery” [42]. A tool was “performative (directive)” when the learner had to fulfill directive tasks (eg, “fill-in-the-blank tests,” multiple- and single choice tests, and assignment tasks). A tool was “performative (guided discovery)” when reasoning, thinking, and the integration of knowledge was required (eg, exploration of different chapters and submodules and decision making during the learning process) [42]. Performative tools could be both, “directive” and “guided discovery,” while “performative,” and “receptive” were mutually exclusive.

### Statistical Analysis

The results were analyzed using descriptive statistics. The attributes (formats, learning styles, content areas, and learning goals) of all tools were calculated, as well as inter- and intragroup differences regarding basic and advanced learner level. With respect to learning styles and content areas, overlaps were taken into consideration. The analysis was conducted using Microsoft Excel.

## Results

### Overview

**Figure 1.**
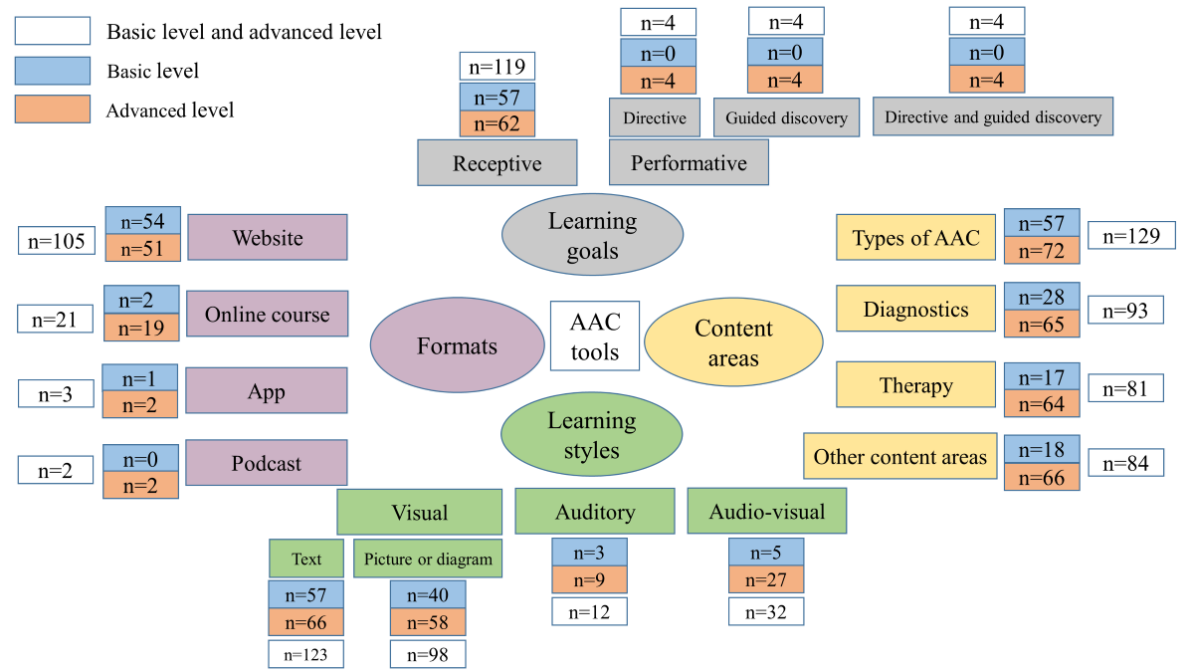
Number of basic level tools and advanced level tools according to formats, content areas, learning styles, and learning goals; n=57 for basic level; n=74 for advanced level. AAC: augmentative and alternative communication.

We identified 131 tools that met the inclusion and exclusion criteria. Multimedia Appendix 4 shows a summary of the list of tools. Of all tools, 43.5% (57/131) were basic level and 56.5% (74/131) were advanced level. Figure 1 shows the number of basic-level tools and advanced-level tools according to formats, content areas, learning styles, and learning goals. The numbers of content areas and learning styles included overlaps (ie, a tool could cover multiple content areas).

### e-Learning Structures Across All Tools

Of all 131 tools, 105 (80.2%) were websites, 21 (16%) were online courses, while only 3 (2.3%) were apps, and 2 (1.5%) were podcasts. In addition, it is worth mentioning that of the 21 online courses, only 2 (9.5%) were in German. Of the 387 content areas including overlaps, 129 (33.3%) pertained to “types of AAC,” 93 (24%) to “diagnostics,” 81 (20.9%) to “therapy,” and 84 (21.7%) to “other content areas.” Of the 265 learning styles including overlaps, “visual (text)” was predominant with 46.4% (123), followed by “visual (picture or diagram) with 37% (98). Only 12.1% (32) were “audio-visual” and only 4.5% (12) were “auditory.” For learning goals, 90.8% (119) of all 131 tools were “receptive,” while only 9.2% (12) were “performative.” Of these 12 performative tools, 4 (33.3%) pertained to “performative (directive),” 4 (33.3%) to “performative (guided discovery),” and 4 (33.3%) to “performative (directive and guided discovery).” Consequently, most of the tools were websites, taught about the types of AAC, showed the “visual (text)” learning style, and had a receptive learning goal. Multimedia Appendix 5 illustrates the distribution of formats, content areas, learning styles, and learning goals across all tools.

### Within-Learner Level Analysis

#### Basic Level

Within all 57 basic level tools, 54 (94.7%) were websites, 2 (3.5%) were online courses, only 1 (1.8%) was an app and none was a podcast. The total number of content areas including overlaps was 120. Of these 120 counts, 57 (47.5%) pertained to “types of AAC,” 28 (23.3%) to “diagnostics,” 17 (14.2%) to “therapy,” and 18 (15%) to “other content areas.” The total number of learning styles including overlaps was 105. Of these 105 counts, 57 (54.3%) were “visual (text),” 40 (38.1%) were “visual (picture or diagram),” 5 (4.8%) were “audio-visual,” and 3 (2.9%) were “auditory.” As for learning goals, all basic-level tools were “receptive” and none were “performative. In conclusion, most of the basic level tools were websites, taught about the “types of AAC,” showed a “visual (text)” learning style, and all of them were "receptive" (Table 1).

**Table 1.** Distribution of augmentative and alternative communication e-learning tools in basic and advanced level according to formats, content areas, learning styles, and learning goals.

	Basic level	Advanced level
Formats		
Formats, n	57	74
Websites, n (%)	54 (94.7)	51 (68.9)
Online courses, n (%)	2 (3.5)	19 (25.7)
Apps, n (%)	1 (1.8)	2 (2.7)
Podcasts, n (%)	0 (0)	2 (2.7)
Content areas		
Content areas, n	120	267
Types of augmentative and alternative communication, n (%)	57 (47.5)	72 (27)
Diagnostics, n (%)	28 (23.3)	65 (24.3)
Therapy, n (%)	17 (14.2)	64 (24)
Other content areas, n (%)	18 (15)	66 (24.7)
Learning styles		
Learning styles, n	105	160
Visual (text), n (%)	57 (54.3)	66 (41.3)
Visual (picture or diagram), n (%)	40 (38.1)	58 (36.3)
Audio-visual, n (%)	5 (4.8)	27 (16.9)
Auditory, n (%)	3 (2.9)	9 (5.6)
Learning goals		
Learning goals, n	57	74
Receptive, n (%)	57 (100)	62 (83.8)
Performative (directive), n (%)	0 (0)	4 (5.4)
Performative (guided discovery), n (%)	0 (0)	4 (5.4)
Performative (directive and guided discovery), n (%)	0 (0)	4 (5.4)

#### Advanced Level

Of the 74 advanced-level tools, 51 (68.9%) were websites, 19 (25.7%) were online courses, 2 (2.7%) were apps, and 2 (2.7%) were podcasts. The total count of content areas including overlaps was 267. Of these 267 counts, 72 (27%) belonged to “types of AAC,” 65 (24.3%) to “diagnostics,” 64 (24%) to “therapy,” and 66 (24.7%) to “other content areas.” The total number of learning styles including overlaps was 160. Of these 160 counts, 66 (41.3%) were “visual (text),” 58 (36.3%) were “visual (picture or a diagram),” 27 (16.9%) were “audio-visual,” and only 9 (5.6%) were “auditory.” Within the 74 advanced-level tools, 62 (83.8%) were “receptive,” 4 (5.4%) required directive tasks, 4 (5.4%) offered a guided discovery, and 4 (5.4%) were directive and offered a guided discovery. To summarize, most of the advanced tools were websites, the content areas were evenly distributed, “visual (text)” was the predominant learning style, and most of the tools were “receptive” (Table 1)

### Between-Learner Level Analysis

We investigated between-learner level differences in formats, content areas, learning styles, and learning goals. To compare basic and advanced level, the higher number of advanced level tools (respectively smaller number of basic level tools) had to be taken into account (57 basic and 74 advanced). Therefore, the absolute numbers cannot be compared. Instead, the percentages (of each component, eg, “website”) within learner levels were compared. In the following paragraphs, the results are presented as “basic level versus advanced level" when results for both learner levels appear in parentheses.

#### Formats

Websites were more common in basic tools (54/57, 94.7% vs 51/74, 68.9%), whereas online courses were more common in advanced tools (2/57, 3.5% vs 19/74, 25.7%). Apps were slightly more common in advanced tools (1/57, 1.8% vs 2/74, 2.7%). Podcasts only appeared in advanced tools (0/57, 0% vs 2/74, 2.7%). In conclusion, websites were more common in basic level, whereas online courses, apps, and podcasts appeared more often in advanced level (Multimedia Appendix 6).

#### Content Areas

All basic level tools and almost all advanced level tools covered “types of AAC” (57/57, 100% vs 72/74, 97.3%). Diagnostic-related content was much more common in advanced level tools (28/57, 49.1% vs 65/74, 87.8%) as were “other content areas” (18/57, 31.6% vs 66/74, 89.2%) and therapeutic-related content (17/57, 29.8% vs 64/74, 86.5%). Advanced level tools were more likely to cover multiple content areas, while showing more diagnostic- and therapy-related content as well as more other content areas (Multimedia Appendix 7).

#### Learning Styles

All basic level tools and most of the advanced level tools had a learning style pertaining to “visual (text)” (57/57, 100% vs 66/74, 89.2%). “Visual (picture or diagram)” was more common in advanced level (40/57, 70.2% vs 58/74 78.4%), as were “audio-visual” (5/57, 8.8% vs 27/74, 36.5%) and “auditory” (3/57, 5.3% vs 9/74, 12.2%) learning styles. Consequently, “visual (picture or diagram),” “auditory,” and “audio-visual” learning styles were more common in advanced level (Multimedia Appendix 8).

#### Learning Goals

All basic level tools and the majority of the advanced level tools were receptive (57/57, 100% vs 62/74, 83.8%). Respectively, basic level tools did not require performance whereas some of the advanced tools did (0/57, 0% vs 12/74, 16.2%) (Table 1).

## Discussion

### Principal Results

Our study set out to explore e-learning tools in AAC for phoniatrics and SLP, analyze their e-learning features and investigate differences in basic and advanced learner level. We identified 131 free access e-learning AAC tools and gained insight into their formats, content areas, learning styles, and learning goals. Most of the tools were websites, while apps and podcasts were rare. The predominant content area was “types of AAC” and “visual (text)” was the most common learning style. Most of the tools were “receptive.” Within both learner levels, “website” was the predominant format. Within basic level, none of the tools were podcasts and the predominant content was “types of AAC.” Within advanced level, the content areas were almost evenly distributed. “Visual (text)” was the predominant learning style in both learner levels. Most of the advanced tools and all basic level tools were “receptive.” Websites were more common in basic level, whereas online courses, apps, and podcasts appeared more in advanced level. The content of advanced level tools was more diagnostic-related and therapy-related. “Visual (picture or diagram),” “auditory,” and “audio-visual” learning styles were more common in advanced level. All basic-level tools and the majority of the advanced-level tools were receptive.

### Interpretation

#### Number of Tools

The relatively large number of 131 tools is encouraging, given that AAC is not yet fully implemented in the curricula of phoniatrics and SLP (at least not in Germany). This number of tools was a good sample size for our analysis. In addition, the number of tools indicates that AAC appears to be a topic of interest in e-learning. This supports a recent study by Burgio [43], who claim that new AAC e-learning tools evolve constantly.

#### Formats

It is not surprising that websites were the predominant format for both learner levels due to the web-based nature of this study. However, we were surprised about the rare number of apps that met our inclusion and exclusion criteria. We found a fair amount of AAC-related apps. However, almost all apps functioned as a communication aid (eg, talker) while lacking a definition of AAC, therefore not being an e-learning tool. Other researchers dealt with these kinds of AAC apps [44]. Unanticipated was the relatively high number of online courses. This supports our claim that AAC is a topic of interest in e-learning. If we had not restricted the inclusion to “free access,” we might have analyzed even more online courses. The fact that most of the online courses pertained to advanced level is in the nature of the thing. Since almost all online courses covered more than just general information, they were assigned to “advanced level.” That made us question our learner level criteria. We could have set the boundary between basic level and advanced level differently (eg, only very detailed tools would be considered as advanced level) or formulated a third category (eg, “intermediate”). That only 2 of the 21 online courses were in German indicates that German-speaking countries lag behind English-speaking countries when it comes to e-learning in AAC. Nevertheless, it is obvious that English (as the predominant language worldwide) is used more often in teaching. The small number of podcasts can be explained by not having specifically searched for podcasts in the first place. Overall, AAC e-learning tools exist in various formats, which indicates that AAC is a topic of interest in e-learning, however, more so in English rather than in German.

#### Content Areas

It is not surprising that advanced level tools covered more content areas. The more detailed the content, the more likely was a tool assigned to advanced level. Again, this might question our learner-level criteria. However, it is interesting that the contents were almost evenly distributed in advanced level, whereas basic level tools covered more “types of AAC” content. Notwithstanding, it seems logical that general information is about the types of AAC. Therapeutic- and diagnostics-related content have a more “advanced” attribute. What do these results mean? We interpret that “types of AAC” seems to be a “basic” content or “general information” that is essential for learners with no previous knowledge of AAC. Information on the types of AAC seems to be an essential content of AAC e-learning tools and should therefore be considered in the development of future modules.

#### Learning Styles

As expected, the predominant learning style was “visual (text),” given that 80.2% of all tools were websites. The fact that online courses and podcasts were more likely in advanced level explains the prevalence of “auditory” and “audio-visual” learning styles in this category. These findings may be somewhat limited by the fact that we had not specifically searched for podcasts. However, it is still interesting to note that audio files and podcasts were more common in advanced level.

#### Learning Goals

In terms of learning goals, we found performative tools only in advanced level. In hindsight, this is obvious. Tools that exceed general information (respectively “advanced level”), more likely required active sensemaking and reasoning. However, we were surprised about the overall small number of performative tools. We therefore suggest that new AAC e-learning tools should offer performative tasks to fill this gap.

### Comparison to Previous Work

This study appears to be the first to investigate the structures of AAC e-learning tools in English and German. In designing our study, we were inspired by Lin and Neuschaefer-Rube [38] who analyzed e-learning tools for SLP, phoniatrics, and otolaryngology by their e-learning structures. Although they set slightly different criteria, their overall idea of investigating e-learning tools can be compared with our study. In their study, for example, learner levels were classified into “academic level” and “clinical professional level.” We chose a different classification for our study, since it cannot be assumed that all clinical professionals have previous knowledge in AAC. Most of our results reflect those of Lin and Neuschaefer-Rube [38] who also found that visual tools were predominant as well as most of the tools were receptive. The difference between the studies was, interestingly, that performative tools pertained more to academic level learners.

The comparison of our findings to yet another study turned out to be challenging. e-Learning studies increased since the COVID-19 pandemic resulting in numerous available research papers. However, other works in the fields of otolaryngology and SLP focused on surveys [45,46], the effectiveness of e-learning [47] or specific online programs [48,49], rather than exploring and analyzing the attributes of e-learning tools. Therefore, our study is a specific examination in the overall bewildering and evolving field of e-learning research.

### Limitations

The findings of this study must be seen in light of 5 limitations. These are (1) incompleteness and difficult replication, (2) limited search terms and search engines, (3) strict inclusion criteria, (4) binary learner level categorization, and (5) quantitative assessment rather than quality rating.

First, e-learning tools are not static since the World Wide Web is an ongoing field of updates and changes [50]. Therefore, this study is only a snapshot of the available tools at the time of data collection undergoing certain inclusion and exclusion criteria. In addition, different IP addresses may lead to different search results. Accordingly, this study is not an investigation of all existing e-learning tools in AAC nor is it replicable. Nevertheless, with a total number of 131 tools, we managed to investigate a high amount, which appears to be a fair representation of the present AAC e-learning scope.

Second, in terms of our web-based search, we could have used more search engines to possibly find more tools [51]. Likewise, we could have added more search terms. Nonetheless, our chosen search engines and search terms led to many results that would not have been manageable without strict inclusion criteria.

Third, the inclusion criterium of a mandatory definition of AAC may have eliminated some advanced tools. In fact, the authors cannot recollect that this was the case during the process of finding the tools. Nevertheless, if we had included tools that did not provide a definition of AAC, probably almost every website within the search results would have entered our study.

Fourth, the binary categorization into either “basic level” or “advanced level” did not leave room for learners with knowledge in between those levels (eg, intermediate level). However, we think that this categorization allows students and clinical professionals to choose a tool according to their knowledge level rather than to their professional status.

Finally, this quantitative study cannot give quality evaluations of the 131 tools, nor do we have proof that the authors of the tools were professionals or well versed in AAC. However, since we investigated the content of the tools, we can conclude that all tools seemed to provide correct information. Despite of its limitations, our study certainly adds to the understanding of e-learning in AAC.

### Future Directions

Our study lays the groundwork for future research in e-learning in AAC. More work needs to be done to gain further insight into e-learning in AAC, such as (1) expanding the analysis to other formats, (2) assessing the quality of the tools, (3) conducting a survey study on e-learning in AAC, and (4) developing new AAC e-learning tools.

First, the analysis of AAC e-learning tools on video sharing platforms, social networks, blogs, and forums would be interesting, since we excluded them in our study. Furthermore, an explicit search for a particular format (eg, podcasts) could lead to more results that might be worth investigating. In addition, an assessment of misinformation on AAC in social media would be informative.

Second, a qualitative analysis of AAC e-learning tools would be beneficial for the users of these tools. The best rated tools could be added to a possible future toolbox app [37], or to a German “AAC online learning platform” suggested by Burgio [43]. Furthermore, it would be interesting to investigate correlations between the e-learning structures and the quality of the tools.

Third, a survey study in phoniatrics and SLP could help identify the preferred structures of possible AAC e-learning tools. It would be worth investigating whether phoniatricians and speech-language pathologists have the same demands and would therefore benefit from the same tool.

Finally, we suggest practical applications for the development of new e-learning tools in AAC for students and professionals in phoniatrics and SLP. One example of a possible AAC e-learning tool could be a German, advanced level, audio-visual online course with a knowledge quiz that covers all content areas relevant to phoniatricians and speech-language pathologists. On the basis of this study, we developed such an online course which is currently being tested with students of medicine and SLP at RWTH Aachen University. Another example would be the development of apps that teach AAC (in both languages).

### Conclusion

To the best of our knowledge, our exploratory study marks the beginning of the investigation of e-learning tools in AAC for phoniatrics and SLP. We found a fair number of tools and gained insight into their formats, content areas, learning styles, and learning goals. Our data indicate that e-learning in AAC is a topic of interest and needs to be further investigated. Overall, we established a basis for future research and suggested practical applications for e-learning in AAC.

## Supplementary material

10.2196/63392 Multimedia Appendix 1 PRISMA (Preferred Reporting Items for Systematic Reviews and Meta-Analyses) 2020 checklist.

10.2196/63392 Multimedia Appendix 2 Search protocol.

10.2196/63392 Multimedia Appendix 3 Data abstraction protocol.

10.2196/63392 Multimedia Appendix 4 Summary list of tools.

10.2196/63392 Multimedia Appendix 5 Distribution of attributes across all tools.

10.2196/63392 Multimedia Appendix 6 Formats.

10.2196/63392 Multimedia Appendix 7 Content areas.

10.2196/63392 Multimedia Appendix 8 Learning styles.

## Abbreviations

AAC: augmentative and alternative communication
PRISMA: Preferred Reporting Items for Systematic Reviews and Meta-Analyses
RWTH: Rheinisch-Westfälische Technische Hochschule
SLP: speech-language pathology
