# Supplementary material for: e-Learning in Phoniatrics and Speech-Language Pathology: Exploratory Analysis of Free Access Tools in Augmentative and Alternative Communication
Source: JMIR Med Educ. 2025 Jun 26;11:e63392. doi: 10.2196/63392 (PMC12256706; doi:10.2196/63392)
Supplement: Multimedia Appendix 2 [file mededu-v11-e63392-s002.pdf]

Multimedia Appendix 2, Search Protocol

Date	SE	Search Term (Verbatim)	Records Screened (n=1616)	Tools Included in the Study (n=131)
11.05.2023	G	>“Augmentative and alternative communication” AND e-learning<	1-25	T3, T26, T34, T42, T103, T129
11.05.2023	G	>AAC AND e-learning<	1-25	T15, T42, T103
12.05.2023	G	>“Augmentative and alternative communication” AND “digital learning”<	1-25	-
12.05.2023	G	>AAC AND “digital learning”<	1-25	T18, T131
16.05.2023	G	>“Augmentative and alternative communication” AND webinar<	1-25	T11, T18, T55, T92
16.05.2023	G	>AAC AND webinar<	1-25	T11, T18, T55, T92
10.05.2023	G	>“What is augmentative and alternative communication”<	1-25	T13, T18, T23, T33, T38, T39, T51, T70, T74, T80, T82, T86, T90, T109, T111, T119, T128, T130
10.05.2023	G	>“What is AAC”<	1-25	T2, T12, T15, T16, T18, T26, T55, T86, T71, T106, T108, T111
12.05.2023	G	>“Augmentative and alternative communication”<	1-25	T12, T14, T15, T26, T33, T43, T55, T77, T80, T90, T121, T122, T130
12.05.2023	G	>AAC<	1-25	T14, T15, T43, T86
09.05.2023	G	>“Unterstützte Kommunikation“ AND E-Learning<	1-25	-
09.05.2023	G	>“Unterstützte Kommunikation“ AND „digitales Lernen“<	1-25	-
16.05.2023	G	>“Unterstützte Kommunikation“ AND Webinar<	1-25	T43, T61, T88, T105, T108, T113
31.05.2023	G	>“Was ist unterstützte Kommunikation“<	1-25	T10, T19, T24, T43, T44, T50, T52, T53, T58, T60, T64, T66, T69, T88, T89, T108, T112, T113, T114
12.05.2023	G	>“Unterstützte Kommunikation“<	1-25	T19, T27, T41, T43, T47, T56, T61, T65, T67, T88, T89, T130
15.05.2023	G	>“Augmentative and alternative communication” AND organization<	1-10	T14, T55, T114, T130
15.05.2023	G	>AAC AND organization<	1-10	
15.05.2023	G	>“Augmentative and alternative communication” AND organisation<	1-10	T18, T26, T74, T83, T122
15.05.2023	G	>AAC AND organisation<	1-10	
15.05.2023	G	>“Augmentative and alternative communication” AND society<	1-10	T14, T43, T55, T74, T127, T130
15.05.2023	G	>AAC AND society<	1-10	
15.05.2023	G	>“Augmentative and alternative communication” AND association<	1-10	T8, T14, T33
15.05.2023	G	>AAC AND association<	1-10	
16.05.2023	G	>“Augmentative and alternative communication” AND club<	1-10	T54
16.05.2023	G	>AAC AND club<	1-10	
16.05.2023	G	>“Augmentative and alternative communication” AND charity<	1-10	T14, T26, T51, T74, T77, T90
16.05.2023	G	>AAC AND charity<	1-10	
16.05.2023	G	>“Augmentative and alternative communication” AND foundation<	1-10	T14, T32, T77, T78
16.05.2023	G	>AAC AND foundation<	1-10	
17.05.2023	G	>“Augmentative and alternative communication” AND initiative<	1-10	T55, T98, T123
17.05.2023	G	>AAC AND initiative<	1-10	
22.05.2023	G	>“Augmentative and alternative communication” AND trust<	1-10	T26, T77
22.05.2023	G	>AAC AND trust<	1-10	
16.05.2023	G	>“Augmentative and alternative communication” AND university<	1-10	T14, T113, T118, T119, T121, T122
16.05.2023	G	>AAC AND university<	1-10	

Multimedia Appendix 2, Search Protocol

16.05.2023	G	>“Augmentative and alternative communication” AND college<	1-10	T3, T90, T119
16.05.2023	G	>AAC AND college<	1-10	
16.05.2023	G	>“Augmentative and alternative communication” AND academy<	1-10	T14, T103
16.05.2023	G	>AAC AND academy<	1-10	
16.05.2023	G	>“Augmentative and alternative communication” AND “health center”<	1-10	T9, T93
16.05.2023	G	>AAC AND “health center”<	1-10	
16.05.2023	G	>“Augmentative and alternative communication” AND “health centre”<	1-10	T23, T77
16.05.2023	G	>AAC AND “health centre”<	1-10	
22.05.2023	G	>“Augmentative and alternative communication” AND “advice center”<	1-10	T87
22.05.2023	G	>AAC AND “advice center”<	1-10	
22.05.2023	G	>“Augmentative and alternative communication” AND “advice centre”<	1-10	T26, T71, T108
22.05.2023	G	>AAC AND “advice centre”<	1-10	
16.05.2023	G	>“Augmentative and alternative communication” AND “established SLP”<	1-10	-
16.05.2023	G	>AAC AND “established SLP”<	1-10	
16.05.2023	G	>“Augmentative and alternative communication” AND hospital<	1-10	T14, T76, T77
16.05.2023	G	>AAC AND hospital<	1-10	
16.05.2023	G	>“Augmentative and alternative communication” AND clinic<	1-10	T49, T125, T126
16.05.2023	G	>AAC AND clinic<	1-10	
17.05.2023	G	>“Augmentative and alternative communication” AND consultation<	1-10	T14, T113, T114
17.05.2023	G	>AAC AND consultation<	1-10	
25.05.2023	G	>“Augmentative and alternative communication” AND “special needs” AND school<	1-10	T46, T109
25.05.2023	G	>AAC AND “special needs” AND school<	1-10	
30.05.2023	G	>“Augmentative and alternative communication” AND “special needs” AND “care center”<	1-10	T114, T57, T72
30.05.2023	G	>AAC AND “special needs” AND “care center”<	1-10	
30.05.2023	G	>“Augmentative and alternative communication” AND “special needs” AND “care centre”<	1-10	T26, T57, T122
30.05.2023	G	>AAC AND “special needs” AND “care centre”<	1-10	
30.05.2023	G	>“Augmentative and alternative communication” AND inclusion<	1-10	T14, T113, T108
30.05.2023	G	>AAC AND inclusion<	1-10	
30.05.2023	G	>“Augmentative and alternative communication” AND “self-help”<	1-10	T3
30.05.2023	G	>AAC AND “self-help”<	1-10	
30.05.2023	G	>“Augmentative and alternative communication” AND “peer-support”<	1-10	T14, T12
30.05.2023	G	>AAC AND “peer-support”<	1-10	
30.05.2023	G	>“Augmentative and alternative communication” AND “provider of communication aids”<	1-10	-
30.05.2023	G	>AAC AND “provider of communication aids”<	1-10	
15.05.2023	G	>“Unterstützte Kommunikation” AND Organisation<	1-10	-
15.05.2023	G	>“Unterstützte Kommunikation” AND Gesellschaft<	1-10	T29, T43
15.05.2023	G	>“Unterstützte Kommunikation” AND Verband<	1-10	T28, T43
15.05.2023	G	>“Unterstützte Kommunikation” AND Verein<	1-10	T10, T20, T43, T86, T87

Multimedia Appendix 2, Search Protocol

15.05.2023	G	>“Unterstützte Kommunikation” AND Initiative<	1-10	T29, T43, T47, T86
22.05.2023	G	>“Unterstützte Kommunikation” AND Stiftung<	1-10	T43, T94, T95, T96, T97
15.05.2023	G	>“Unterstützte Kommunikation” AND Universität<	1-10	T113, T114
15.05.2023	G	>“Unterstützte Kommunikation” AND Hochschule<	1-10	-
15.05.2023	G	>“Unterstützte Kommunikation” AND Bildungsportal<	1-10	T48, T87
15.05.2023	G	>“Unterstützte Kommunikation” AND Beratungsstelle<	1-10	T19, T43, T47, T61, T113
15.05.2023	G	>“Unterstützte Kommunikation” AND „logopädische Praxis“<	1-10	T43, T59
15.05.2023	G	>“Unterstützte Kommunikation” AND Klinik<	1-10	T43, T63, T65, T89
15.05.2023	G	>“Unterstützte Kommunikation” AND Krankenhaus<	1-10	T43, T63
15.05.2023	G	>“Unterstützte Kommunikation” AND Förderschule<	1-10	T48, T65, T91
17.05.2023	G	>“Unterstützte Kommunikation” AND Kindertagesstätte<	1-10	T27, T43, T61, T86
15.05.2023	G	>“Unterstützte Kommunikation” AND Werkstatt<	1-10	T30
15.05.2023	G	>“Unterstützte Kommunikation” AND Inklusion<	1-10	T17, T6, T88, T50
15.05.2023	G	>“Unterstützte Kommunikation” AND Selbsthilfe<	1-10	T19, T29, T61
15.05.2023	G	>“Unterstützte Kommunikation” AND Arbeitskreis<	1-10	T43, T61, T65
15.05.2023	G	>“Unterstützte Kommunikation” AND Betroffene<	1-10	T43, T56
15.05.2023	G	>“Unterstützte Kommunikation” AND Hilfsmittelanbieter<	1-10	T29, T43, T58
12.05.2023	GPS	>“Augmentative and alternative communication” AND e-learning<	NR	-
12.05.2023	GPS	>AAC AND e-learning<	NR	-
12.05.2023	GPS	>“Augmentative and alternative communication” AND “digital learning”<	NR	-
12.05.2023	GPS	>AAC AND “digital learning”<	NR	-
12.05.2023	GPS	>“Augmentative and alternative communication” AND webinar<	NR	-
12.05.2023	GPS	>AAC AND webinar<	NR	-
12.05.2023	GPS	>“What is augmentative and alternative communication”<	NR	-
12.05.2023	GPS	>“What is AAC”<	NR	-
12.05.2023	GPS	>“Augmentative and alternative communication”<	1-25	T16, T38, T82
12.05.2023	GPS	>AAC<	1-25	T16, T82
12.05.2023	GPS	>“Unterstützte Kommunikation“ AND E-Learning<	NR	-
12.05.2023	GPS	>“Unterstützte Kommunikation“ AND „digitales Lernen“<	NR	-
12.05.2023	GPS	>“Unterstützte Kommunikation“ AND Webinar<	NR	-
31.05.2023	GPS	>“Was ist unterstützte Kommunikation“<	NR	-
12.05.2023	GPS	>“Unterstützte Kommunikation“<	1-25	T16, T62, T82
12.05.2023	AS	>“Augmentative and alternative communication” AND e-learning<	NR	-
12.05.2023	AS	>AAC AND e-learning<	NR	-
12.05.2023	AS	>“Augmentative and alternative communication” AND “digital learning”<	NR	-
12.05.2023	AS	>AAC AND “digital learning”<	NR	-
12.05.2023	AS	>“Augmentative and alternative communication” AND webinar<	NR	-
12.05.2023	AS	>AAC AND webinar<	NR	-
12.05.2023	AS	>“What is augmentative and alternative communication”<	NR	-
12.05.2023	AS	>“What is AAC”<	NR	-
12.05.2023	AS	>“Augmentative and alternative communication”<	1-25	T38, T82

12.05.2023	AS	>AAC<	1-25	T1, T16, T40, T82
12.05.2023	AS	>“Unterstützte Kommunikation“ AND E-Learning<	NR	-
12.05.2023	AS	>“Unterstützte Kommunikation“ AND „digitales Lernen“<	NR	-
12.05.2023	AS	>“Unterstützte Kommunikation“ AND Webinar<	NR	-
31.05.2023	AS	>“Was ist unterstützte Kommunikation“<	NR	-
12.05.2023	AS	>“Unterstützte Kommunikation“<	1-25	T45, T62
01.06.2023	GS	>“Unterstützte Kommunikation“<	1-25	T113 (not directly found but mentioned)
01.06.2023	GS	>“Unterstützte Kommunikation“ AND E-Learning<	1-25	-
01.06.2023	GS	>“Unterstützte Kommunikation“ AND „digitales Lernen“<	1-25	-
01.06.2023	GS	>“Augmentative and alternative communication”<	1-25	T14 (not directly found but mentioned)
01.06.2023	GS	>AAC<	1-25	-
01.06.2023	GS	>“Augmentative and alternative communication” AND e-learning<	1-25	-
01.06.2023	GS	>“Augmentative and alternative communication” AND “digital learning”<	1-25	-
01.06.2023	PM	>“Unterstützte Kommunikation“<	3	-
01.06.2023	PM	>“Unterstützte Kommunikation“ AND E-Learning<	NR	-
01.06.2023	PM	>“Unterstützte Kommunikation“ AND „digitales Lernen“<	NR	-
01.06.2023	PM	>“Augmentative and alternative communication”<	1-25	T14, T56 (not directly found but mentioned)
01.06.2023	PM	>AAC<	1-25	T14, T56 (not directly found but mentioned)
01.06.2023	PM	>“Augmentative and alternative communication” AND e-learning<	1	-
01.06.2023	PM	>“Augmentative and alternative communication” AND “digital learning”<	NR	-
01.06.2023	PD	>“Unterstützte Kommunikation“<	1-25	T113, T114 (not directly found but mentioned)
01.06.2023	PD	>“Unterstützte Kommunikation“ AND E-Learning<	18	-
01.06.2023	PD	>“Unterstützte Kommunikation“ AND „digitales Lernen“<	7	-
01.06.2023	PD	>“Augmentative and alternative communication”<	1-25	T14 (not directly found but mentioned)
01.06.2023	PD	>AAC<	1-25	-
01.06.2023	PD	>“Augmentative and alternative communication” AND e-learning<	10	-
01.06.2023	PD	>“Augmentative and alternative communication” AND “digital learning”<	2	-
02.06.2023	-	-	-	Tools added by author JB: T5, T35

Abbreviations:

Date: Date of search

SE: Search engine; G = Google; GPS = Google Play Store; AS = Apple App Store; GS = Google Scholar; PM = PubMed; PD = peDOCS

Records Screened: 1-25 = The first 25 search results underwent the screening for in- and exclusion criteria; 1-10 = The first 10 search results underwent the screening for in- and exclusion criteria; NR = None, due to no results; other numbers (eg, 3) = maximum number of search results that all underwent the screening for in- and exclusion criteria

Tools Included in the Study: T1 = AAC Coach; T2 = AAC Institute; T3 = AAC Learning Center Moodle; T4 = ABIOS – Acquired Brain Injury Outreach Service; T5 = AKUK – Arbeitskreis UK; T6 = Alison; T7 = All About AAC; T8 = ALS Association; T9 = American Academy of Pediatrics; T10 = Angelman e. V.; T11 = APSEA – Atlantic Provinces Special Education Authority; T12 = ARASAAC; T13 = Ascend Health; T14 = ASHA – American Speech-Language-Hearing Association; T15 = Assistive Ware; T16 = Avaz inc.; T17 = BPB – Bundeszentrale für Politische Bildung; T18 = CALL Scotland; T19 = Caritas; T20 = CDKL5 Deutschland e. V.; T21 = Center for Autism and Related Disorders; T22 = CFHD – Children & Family Health Devon; T23 = Children’s Treatment Network; T24 = Christophorus Schule; T25 = Classinc.net; T26 = Communication Matters; T27 = Cooperative Mensch; T28 = DBL – Deutscher Bundesverband für Logopädie e. V.; T29 = DGM – Deutsche Gesellschaft für Muskelkranke; T30 = Diakoneo; T31 = Die UK-Kiste; T32 = DSRF – Down Syndrome Resource Foundation; T33 = EBIP – Evidence-Based Instructional Practices; T34 = ECPC – Early Childhood Personnel Center; T35 = Epitech; T36 = Everyone Communicates – The AAC Resource; T37 = Explore AAC; T38 = Fabulaa; T39 = FCPS – Fairfax County Public Schools; T40 = Fluent AAC; T41 = Frühförderstelle Unna; T42 = George Jeffrey Children’s Centre; T43 = Gesellschaft für UK e. V.; T44 = Haldenwang Schule; T45 = HE-App –

Humanelektronik; T46 = Heartspring; T47 = Hegau-Jugendwerk; T48 = Helen-Keller-Schule Wiehl; T49 = Hennick Bridgepoint Hospital; T50 = Hypotheses; T51 = In Focus – The Vision Impairment and Complex Needs Charity; T52 = Inclusion 24; T53 = Intakt.info – Fragen und Antworten zu meinem Kind mit Behinderung; T54 = IOWA College; T55 = ISAAC – International Society of AAC; T56 = JG Heinrich-Haus; T57 = KANAV; T58 = KinderUK – Der Kompaktkurs für UK; T59 = Kölner Praxis für Logopädie und UK; T60 = Landesbildungsserver Baden-Württemberg; T61 = Lebenshilfe; T62 = Lebenswelt; T63 = LogbUK; T64 = Louis Braille Schule; T65 = LVR Landschaftsverband Rheinland; T66 = LWL – Landschaftsverband Westfalen-Lippe; T67 = MARLI GmbH; T68 = Menschen im Mittelpunkt – Ines König; T69 = Mia san dabei! – Rita-Maria Donhauser; T70 = Milestone Clinic; T71 = MNDA – Motor Neurone Disease Association; T72 = NadiIntelek; T73 = NAPA – Neurological and Physical Abiliation Center; T74 = National Autistic Society; T75 = NIDCD – National Institute on Deafness and other Communication Disorders; T76 = Nationwide Children's; T77 = NHS – National Health Service United Kingdom; T78 = NLMF – Nancy Lurie Marks Family Foundation; T79 = NCACA – North Carolina Augmentative Communication Association; T80 = Novita Tech; T81 = NWACS – Northwest Augmentative Communication Society; T82 = Otismo; T83 = Pace; T84 = Patient-Provider Communication; T85 = PaTTAN – Pennsylvania Training and Technical Assistance Network; T86 = PRD – Prentke Romich; T87 = REHADAT; T88 = RehaMedia; T89 = REHAVISTA; T90 = RCSLT – Royal College of Speech and Language Therapists; T91 = Schule am Buschkamp; T92 = Smartbox – Simple AAC; T93 = Stanford Medicine – Children's Health; T94 = Stiftung Eben-Ezer; T95 = Stiftung Haus Hall; T96 = Stiftung Kreuznacher Diakonie; T97 = Stiftung Waldheim; T98 = SWAAC – Statewide Assistive Technology AAC; T99 = Talk Tools; T100 = Talking with Tech – AAC Podcast; T101 = TECH OWL – AAC COMMUNITY; T102 = The Center for AAC & Autism; T103 = The Education People; T104 = The Royal Children's Hospital Melbourne; T105 = Therapie Experte; T106 = Therapy Focus; T107 = To Signify GmbH; T108 = Tobii Dynavox; T109 = Touch-Type Read & Spell; T110 = TW – Therapy Works; T111 = Twinkl; T112 = UK-Schweiz – Geballte Power für UK; T113 = Universität Köln, Forschungs- und Beratungszentrum für UK; T114 = Universität Oldenburg; T115 = University of Alabama; T116 = University of Edinburgh; T117 = University of Manchester; T118 = University of Miami; T119 = University of Nebraska-Lincoln; T120 = University of Niagara, Disability Awareness Training; T121 = University of North Carolina, AFIRM – Autism Focused Intervention; T122 = University of Pretoria; T123 = University of Vanderblit; T124 = University of Washington; T125 = University of Wisconsin-Madison; T126 = University of Ohio, Wexner Medical Center; T127 = USSAAC – United States Society for AAC; T128 = WebMD; T129 = West Park Long-term Ventilation Centre of Excellence; T130 = Wikipedia; T131 = Wisconsin Department of Public Instruction
