# Supplementary material for: e-Learning in Phoniatrics and Speech-Language Pathology: Exploratory Analysis of Free Access Tools in Augmentative and Alternative Communication
Source: JMIR Med Educ. 2025 Jun 26;11:e63392. doi: 10.2196/63392 (PMC12256706; doi:10.2196/63392)
Supplement: Multimedia Appendix 3 [file mededu-v11-e63392-s003.pdf]

Multimedia Appendix 3, Data Abstraction Protocol

**Area of Analysis**	**Instructions for Tool Analysis and Data Collection**<br>Choose a tool from the list. Work through the tool and document its attributes in the table below.
Language	Mark which language is used: German and / or English.
Format	Mark which of the following formats is used (single choice): website, online course, app, or podcast.
Content Areas	Mark which content areas are covered (single or multiple choice): types of AAC, diagnostics, therapy, or other content areas.<br>• Types of AAC: a detailed explanation of at least one type of AAC or an overview of the types of AAC is provided.<br>• Diagnostics: at least one medical condition of AAC users is provided.<br>• Therapy: at least one example of a patient and their type of AAC is provided.<br>• Other content areas: other valuable information is provided such as downloads, glossaries, or description of specific approaches in AAC.
Learning Styles	Mark which learning styles are displayed (single or multiple choice): visual (text), visual (picture or diagram), auditory, and / or audio-visual.<br>• Visual (text): reading a text<br>• Visual (picture or diagram): interpreting diagrams, looking at pictures<br>• Auditory: hearing audio-files<br>• Audio-visual: consuming a video
Learning Goals	Mark which learning goal is to be pursued (receptive or performative).<br>• Receptive: passive consumption (only reading or listening)<br>• Performative: directive tasks had to be fulfilled (“directive”) and / or reasoning, thinking, and the integration of knowledge was required (“guided discovery”)
Learner Level	Decide which learner level the tool is assigned to (basic or advanced).<br>• Basic: tool provides only general information<br>• Advanced: tool exceeds general information, tool requires prior knowledge or clinical experience
Comments	Note information on bias, discussions with co-author, if a link needs to be checked again, or any other comment that facilitates the process.

**Tool**	**Language**	**Format**	**Content Areas**	**Learning Styles**	**Learning Goals**	**Learner Level**	**Comments**
eg, T1	☐ English<br>☐ German	☐ Website<br>☐ Online course<br>☐ App<br>☐ Podcast	☐ Types of AAC<br>☐ Diagnostics<br>☐ Therapy<br>☐ Other content areas	☐ Visual (text)<br>☐ Visual (picture or diagram)<br>☐ Auditory<br>☐ Audio-visual	☐ Receptive<br>☐ Performative<br>☐ Directive<br>☐ Guided discovery	☐ Basic<br>☐ Advanced	☐ Check link again<br>☐ Bias:<br>☐ Discuss with co-author:<br>☐ Other:

Abbreviations:

AAC = Augmentative and alternative communication
