# Supplementary material for: e-Learning in Phoniatrics and Speech-Language Pathology: Exploratory Analysis of Free Access Tools in Augmentative and Alternative Communication
Source: JMIR Med Educ. 2025 Jun 26;11:e63392. doi: 10.2196/63392 (PMC12256706; doi:10.2196/63392)
Supplement: Multimedia Appendix 4 [file mededu-v11-e63392-s004.pdf]

Multimedia Appendix 4, Summary List of Tools

Tool Name	Language	Learner Level	Format	Content Area	Learning Style	Learning Goal	Link
AAC Coach	Eng	Advanced	App	T, D, O	X, PoD	PGD	AAC Coach App
AAC Institute	Eng	Advanced	Website	T, D, I, O	X, PoD	Receptive	AAC Institute
AAC Learning Center Moodle	Eng	Advanced	Onl. course	T, D, I, O	X, PoD, V	PD, PGD	AAC Learning Center Moodle
ABIOS – Acquired Brain Injury Outreach Service	Eng	Advanced	Website	T, D, I	X, PoD	Receptive	ABIOS
AKUK – Arbeitskreis UK	Ger	Advanced	Website	T, D, I, O	X, PoD, V	Receptive	AKUK
Alison	Eng	Advanced	Onl. course	T, D, I, O	X	PD	Alison Course
All About AAC	Eng	Advanced	Website	T, D, I, O	X, PoD	Receptive	All About AAC
ALS Association	Eng	Basic	Website	T, D, I	X	Receptive	ALS Association
American Academy of Pediatrics	Eng	Basic	Website	T, D	X	Receptive	American Academy of Pediatrics
Angelman e. V.	Ger	Basic	Website	T, D, I, O	X, PoD, V	Receptive	Angelman e. V.
APSEA – Atlantic Provinces Special Education Authority	Eng	Advanced	Onl. course	T, D, I, O	X, PoD, V	Receptive	APSEA Course
ARASAAC	Eng, Ger	Advanced	Website	T, D, I, O	X, PoD	Receptive	ARA English ARA German
Ascend Health	Eng	Advanced	Website	T	X, PoD	Receptive	Ascend Health
ASHA – American Speech-Language-Hearing Association	Eng	Advanced	Website	T, D, I, O	X	Receptive	ASHA
Assistive Ware	Eng	Advanced	Website	T, D, I, O	X, PoD	Receptive	Assistive Ware
Avaz inc.	Eng	Basic	Website	T, D	X, PoD	Receptive	Avaz
BPB – Bundeszentrale für Politische Bildung	Ger	Advanced	Website	T, D, O	V	Receptive	BPB
CALL Scotland	Eng	Advanced	Website	T, D, I, O	X, V	Receptive	CALL Scotland
Caritas	Ger	Basic	Website	T, D	X, PoD	Receptive	Caritas
CDKL5 Deutschland e. V.	Ger	Basic	Website	T, D	X, PoD	Receptive	CDLK5
Center for Autism and Related Disorders	Eng	Advanced	Onl. course	T, D, I, O	X, PoD	Receptive	Center for Autism
CFHD – Children & Family Health Devon	Eng	Basic	Website	T	X	Receptive	CFHD
Children’s Treatment Network	Eng	Basic	Onl. course	T, D, O	X, PoD, V	Receptive	Children's Treatment Network
Christophorus Schule	Ger	Basic	Website	T	X, PoD	Receptive	Christophorus Schule
Classinc.net	Eng	Advanced	Website	T, D, I, O	X, PoD	Receptive	Classinc
Communication Matters	Eng	Advanced	Website	T, D, I, O	X, PoD	Receptive	Communication Matters

Multimedia Appendix 4, Summary List of Tools

Cooperative Mensch	Ger	Advanced	Website	T, D, O	X, PoD	Receptive	Cooperative Mensch
DBL – Deutscher Bundesverband für Logopädie e. V.	Ger	Basic	Website	T, D, I	X, PoD	Receptive	DBL
DGM – Deutsche Gesellschaft für Muskelkranke	Ger	Advanced	Website	T, D, I, O	X, PoD	Receptive	DGM
Diakoneo	Ger	Basic	Website	T, D, I	X, PoD	Receptive	Diakoneo
Die UK-Kiste	Ger	Advanced	Website	T, D, I, O	X, PoD	Receptive	UK-Kiste
DSRF – Down Syndrome Resource Foundation	Eng	Advanced	Podcast	T, D, I, O	A	Receptive	DSRF Podcast
EBIP – Evidence-Based Instructional Practices	Eng	Advanced	Website	T, D, I, O	X, PoD, V	Receptive	EBIP
ECPC – Early Childhood Personnel Center	Eng	Basic	Onl. course	T, O	X, PoD	Receptive	ECPC Course
Epitech	Ger	Advanced	Website	T, D, I, O	X, PoD	Receptive	Epitech
Everyone Communicates – The AAC Resource	Eng	Advanced	Website	T, D, I, O	X	Receptive	Everyone Communicates
Explore AAC	Eng	Advanced	Onl. course	T, D, I, O	A, X, PoD, V	PD, PGD	Explore AAC
Fabulaa	Eng	Basic	Website	T, D, O	X, PoD	Receptive	Fabulaa
FCPS – Fairfax County Public Schools	Eng	Advanced	Onl. course	T, D, I, O	A, X, PoD, V	Receptive	FCPS
Fluent AAC	Eng	Advanced	Website	T, D, O	X, PoD	Receptive	Fluent AAC
Frühförderstelle Unna	Ger	Basic	Website	T, I, O	X, PoD, V	Receptive	FF Unna
George Jeffrey Children’s Centre	Eng	Advanced	Onl. course	T, I, O	X, PoD	PD	GJ Children's Centre Course
Gesellschaft für UK e. V.	Ger	Advanced	Website	T, D, I, O	X, PoD, V	Receptive	GesUK
Haldenwang Schule	Ger	Advanced	Website	T, I, O	X, PoD	Receptive	Haldenwang Schule
HE-App – Humanelektronik	Ger	Basic	App	T, I, O	X, PoD	Receptive	HE-App
Heartspring	Eng	Advanced	Onl. course	T, I, O	V	Receptive	Heartspring
Hegau-Jugendwerk	Ger	Basic	Website	T	X, PoD	Receptive	Hegau-Jugendwerk
Helen-Keller-Schule Wiehl	Ger	Advanced	Website	T, I, O	X, PoD	Receptive	Helen-Keller-Schule
Hennick Bridgepoint Hospital	Eng	Basic	Website	T	X	Receptive	HB Hospital
Hypotheses	Ger	Advanced	Website	T, D, I, O	A, X, PoD	Receptive	Hypotheses
In Focus – The Vision Impairment and Complex Needs Charity	Eng	Basic	Website	T, D	X, PoD	Receptive	In Focus
Inclusion 24	Ger	Basic	Website	T	X	Receptive	Inclusion 24
Intakt.info – Fragen und Antworten zu meinem Kind mit Behinderung	Ger	Advanced	Website	T, D, I, O	X	Receptive	Intakt
IOWA College	Eng	Basic	Website	T, D, I, O	X, PoD	Receptive	IOWA College

Multimedia Appendix 4, Summary List of Tools

ISAAC – International Society of AAC	Eng	Advanced	Website	T, D, I, O	X, PoD	Receptive	ISAAC
JG Heinrich-Haus	Ger	Basic	Website	T	X, PoD	Receptive	JG Heinrich Haus
KANAV	Eng	Basic	Website	T, I	X, PoD	Receptive	KANAV
KinderUK – Der Kompaktkurs für UK	Ger	Advanced	Onl. course	T, D, I, O	X, V	Receptive	KinderUK Kompaktkurs
Kölner Praxis für Logopädie und UK	Ger	Advanced	Website	T, D, I, O	X, PoD	Receptive	Kölner Praxis
Landesbildungsserver Baden-Württemberg	Ger	Basic	Website	T	X, PoD, V	Receptive	Landesbildungsserver BW
Lebenshilfe	Ger	Basic	Website	T, O	A, X, PoD	Receptive	Lebenshilfe
Lebenswelt	Ger	Advanced	App	T, D, O	X, PoD, V	PGD	Lebenswelt App
LogbUK	Ger	Advanced	Website	T, D, I, O	X, PoD	Receptive	LogbUK
Louis Braille Schule	Ger	Advanced	Website	T, D, I, O	X, PoD	Receptive	Louis Braille Schule
LVR Landschaftsverband Rheinland	Ger	Basic	Website	T, D, I, O	X, PoD	Receptive	LVR
LWL – Landschaftsverband Westfalen-Lippe	Ger	Basic	Website	T	A, X, PoD	Receptive	LWL
MARLI GmbH	Ger	Basic	Website	T, D	A, X, PoD	Receptive	MARLI
Menschen im Mittelpunkt – Ines König	Ger	Basic	Website	T, O	X	Receptive	Menschen im Mittelpunkt
Mia san dabei! – Rita-Maria Donhauser	Ger	Basic	Website	T	X, PoD	Receptive	Mia san dabei
Milestone Clinic	Eng	Advanced	Website	T, D, I	X, PoD	Receptive	Milestone Clinic
MNDA – Motor Neurone Disease Association	Eng	Advanced	Website	T, D, I, O	X, PoD	Receptive	MNDA
NadiIntelek	Eng	Basic	Website	T	X, PoD	Receptive	NadiIntelek
NAPA – Neurological and Physical Abiliation Center	Eng	Advanced	Website	T, D, I, O	X, PoD	Receptive	NAPA
National Autistic Society	Eng	Advanced	Website	T, D, I	X	Receptive	National Autistic Society
NIDCD – National Institute on Deafness and other Communication Disorders	Eng	Advanced	Website	T, D	X, PoD	Receptive	NIDCD
Nationwide Children's	Eng	Basic	Website	T, D, O	X	Receptive	Nationwide Children's
NHS – National Health Service United Kingdom	Eng	Advanced	Website	T, D, I, O	X, PoD, V	Receptive	NHS
NLMF – Nancy Lurie Marks Family Foundation	Eng	Basic	Website	T, D	X	Receptive	NLMF Foundation
NCACA – North Carolina Augmentative Communication Association	Eng	Basic	Website	T, O	X, PoD	Receptive	NCACA
Novita Tech	Eng	Advanced	Website	T, I, O	X, PoD	Receptive	Novita Tech
NWACS – Northwest Augmentative Communication Society	Eng	Advanced	Website	T, D, I, O	X, PoD, V	Receptive	NWACS
Otismo	Eng	Basic	Website	T, D	X, PoD	Receptive	Otismo

Pace	Eng	Basic	Website	T, D, I, O	X, PoD, V	Receptive	Pace	
Patient-Provider Communication	Eng	Advanced	Website	T, D, I, O	X, PoD, V	Receptive	Patient-Provider Communication	
PaTTAN – Pennsylvania Training and Technical Assistance Network	Eng	Advanced	Onl. course	T, O	X, PoD, V	Receptive	PaTTAN Course	
PRD – Prentke Romich	Eng, Ger	Advanced	Website	T, D, I, O	A, X, PoD, V	Receptive	Prentke English	Prenkte German
REHADAT	Ger	Basic	Website	T, D, O	X	Receptive	REHADAT	
RehaMedia	Ger	Advanced	Website	T, D, I, O	X, PoD	Receptive	RehaMedia	
REHAVISTA	Ger	Advanced	Website	T, D, I, O	X, PoD	Receptive	REHAVISTA	
RCSLT – Royal College of Speech and Language Therapists	Eng	Basic	Website	T	X	Receptive	RCSLT	
Schule am Buschkamp	Ger	Basic	Website	T	X	Receptive	Schule am Buschkamp	
Smartbox – Simple AAC	Eng	Advanced	Onl. course	T, O	V	Receptive	Smartbox Course	
Stanford Medicine – Children’s Health	Eng	Basic	Website	T, D, I	X	Receptive	Standford Children's	
Stiftung Eben-Ezer	Ger	Basic	Website	T	X, PoD	Receptive	Stiftung EE	
Stiftung Haus Hall	Ger	Advanced	Website	T, D, I, O	X, PoD, V	Receptive	Stiftung Haus Hall	
Stiftung Kreuznacher Diakonie	Ger	Basic	Website	T	X, PoD	Receptive	Stiftung K Diakonie	
Stiftung Waldheim	Ger	Basic	Website	T, I	X, PoD	Receptive	Stiftung Waldheim	
SWAAC – Statewide Assistive Technology AAC	Eng	Advanced	Website	I, O	X, PoD	Receptive	SWAAAC	
Talk Tools	Ger	Advanced	Website	T, D, I, O	X, PoD	Receptive	Talk Tools	
Talking with Tech – AAC Podcast	Eng	Advanced	Podcast	T, D, I, O	A	Receptive	Talking With Tech AAC Podcast	
TECH OWL – AAC COMMUNITY	Eng	Advanced	Onl. course	T, D, I, O	A, X, PoD, V	PD	TECH OWL Course	
The Center for AAC & Autism	Eng	Advanced	Website	T, D, I, O	X, PoD	Receptive	The Center for AAC and Autism	
The Education People	Eng	Advanced	Onl. course	T, D, I, O	X, PoD	PD, PGD	The Education People Course	
The Royal Children’s Hospital Melbourne	Eng	Advanced	Website	T, D, I	X	Receptive	RCH Melbourne	
Therapie Experte	Ger	Advanced	Onl. course	T, D, I, O	V	Receptive	Therapieexperte Webinar	
Therapy Focus	Eng	Basic	Website	T, I	X, PoD	Receptive	Therapy Focus	
To Signify GmbH	Ger	Advanced	Website	T, D	X, PoD	Receptive	To Signify	
Tobii Dynavox	Eng, Ger	Basic	Website	T, I, O	X, PoD	Receptive	Tobii German	Tobii English
Touch-Type Read & Spell	Eng	Advanced	Website	T, D, I	X, PoD	Receptive	Touch Type R&S	
TW – Therapy Works	Eng	Basic	Website	T, D	X, PoD	Receptive	Therapy Works	

Multimedia Appendix 4, Summary List of Tools

Twinkl	Eng	Basic	Website	T, O	X, PD	Receptive	Twinkl	
UK-Schweiz – Geballte Power für UK	Ger	Basic	Website	T	X, PD	Receptive	UK Schweiz	
Universität Köln, Forschungs- und Beratungszentrum für UK	Ger	Advanced	Website	T, I, O	A, X, PoD, V	Receptive	Uni Köln	
Universität Oldenburg	Ger	Basic	Website	T, D	X, PoD	Receptive	Uni Oldenburg	
University of Alabama	Eng	Basic	Website	T, I	X, PoD	Receptive	Uni Alabama	
University of Edinburgh	Eng	Advanced	Onl. course	T, D, I, O	A, X, PoD, V	PD	Uni Edinburgh Course	
University of Manchester	Eng	Advanced	Onl. course	T, D, I	X, PoD	PGD	Uni Manchester Course	
University of Miami	Eng	Basic	Website	T	X	Receptive	Uni Miami	
University of Nebraska-Lincoln	Eng	Advanced	Website	D, I, O	X, PoD	Receptive	Uni Nebraska	
University of Niagara, Disability Awareness Training	Eng	Advanced	Website	T, D, O	X, PoD	Receptive	Uni Niagara	
University of North Carolina, AFIRM – Autism Focused Intervention	Eng	Advanced	Onl. course	T, D, I, O	X, PoD, V	PGD	Uni North Carolina Course	
University of Pretoria	Eng	Basic	Website	T, D	X, PoD	Receptive	Uni Pretoria	
University of Vanderblit	Eng	Basic	Website	T, D, I, O	X	Receptive	Uni Vanderblit	
University of Washington	Eng	Basic	Website	T, D	X	Receptive	Uni Washington	
University of Wisconsin-Madison	Eng	Basic	Website	T, D	X, PoD	Receptive	Uni Madison	
University of Ohio, Wexner Medical Center	Eng	Basic	Website	T, D, I	X	Receptive	Uni Ohio	
USSAAC – United States Society for AAC	Eng	Advanced	Website	T, D, I, O	X, PoD, V	Receptive	USSAC	
WebMD	Eng	Basic	Website	T, D, O	X	Receptive	WebMD	
West Park Long-term Ventilation Centre of Excellence	Eng	Advanced	Onl. course	T, D, I, O	V	PD, PGD	Westpark LTVC Course	
Wikipedia	Eng, Ger	Advanced	Website	T, D, I, O	X, PoD	Receptive	Wiki English	Wiki German
Wisconsin Department of Public Instruction	Eng	Advanced	Onl. course	T, D, I, O	V	Receptive	Wisconsin DPI	

Abbreviations:

AAC = Augmentative and alternative communication

UK = Unterstützte Kommunikation (German AAC)

Language: Eng = English; Ger = German

Format: Onl. course = Online course

Content Area: T = Types of AAC; D = Diagnostics; I = Therapy; O = Other content areas

Learning Style: A = Auditory; X = Visual (text); PoD = Visual (picture or diagram); V = Audio-visual

Learning Goal: PD = Performative (directive); PGD = Performative (guided discovery)

Link: These links were active on August 13st, 2023
