# Supplementary material for: e-Learning in Phoniatrics and Speech-Language Pathology: Exploratory Analysis of Free Access Tools in Augmentative and Alternative Communication
Source: JMIR Med Educ. 2025 Jun 26;11:e63392. doi: 10.2196/63392 (PMC12256706; doi:10.2196/63392)
Supplement: Multimedia Appendix 5 [file mededu-v11-e63392-s005.docx]

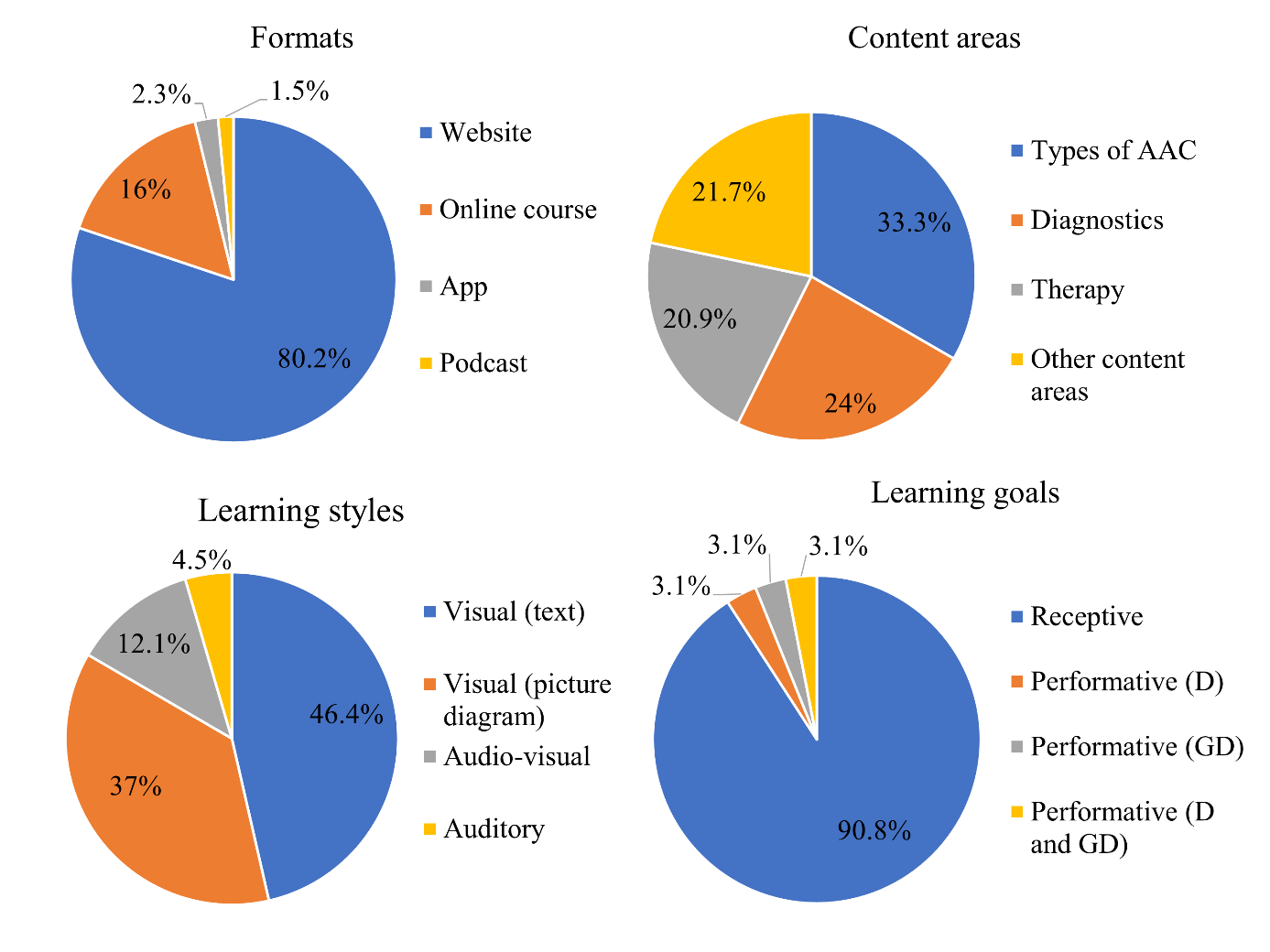


Distribution of formats, content areas, learning styles, and learning goals across all augmentative and alternative communication tools; AAC = augmentative and alternative communication; D = directive; GD = guided discovery; picture diagram = picture or diagram
