# Supplementary material for: e-Learning in Phoniatrics and Speech-Language Pathology: Exploratory Analysis of Free Access Tools in Augmentative and Alternative Communication
Source: JMIR Med Educ. 2025 Jun 26;11:e63392. doi: 10.2196/63392 (PMC12256706; doi:10.2196/63392)
Supplement: Multimedia Appendix 8 [file mededu-v11-e63392-s008.docx]

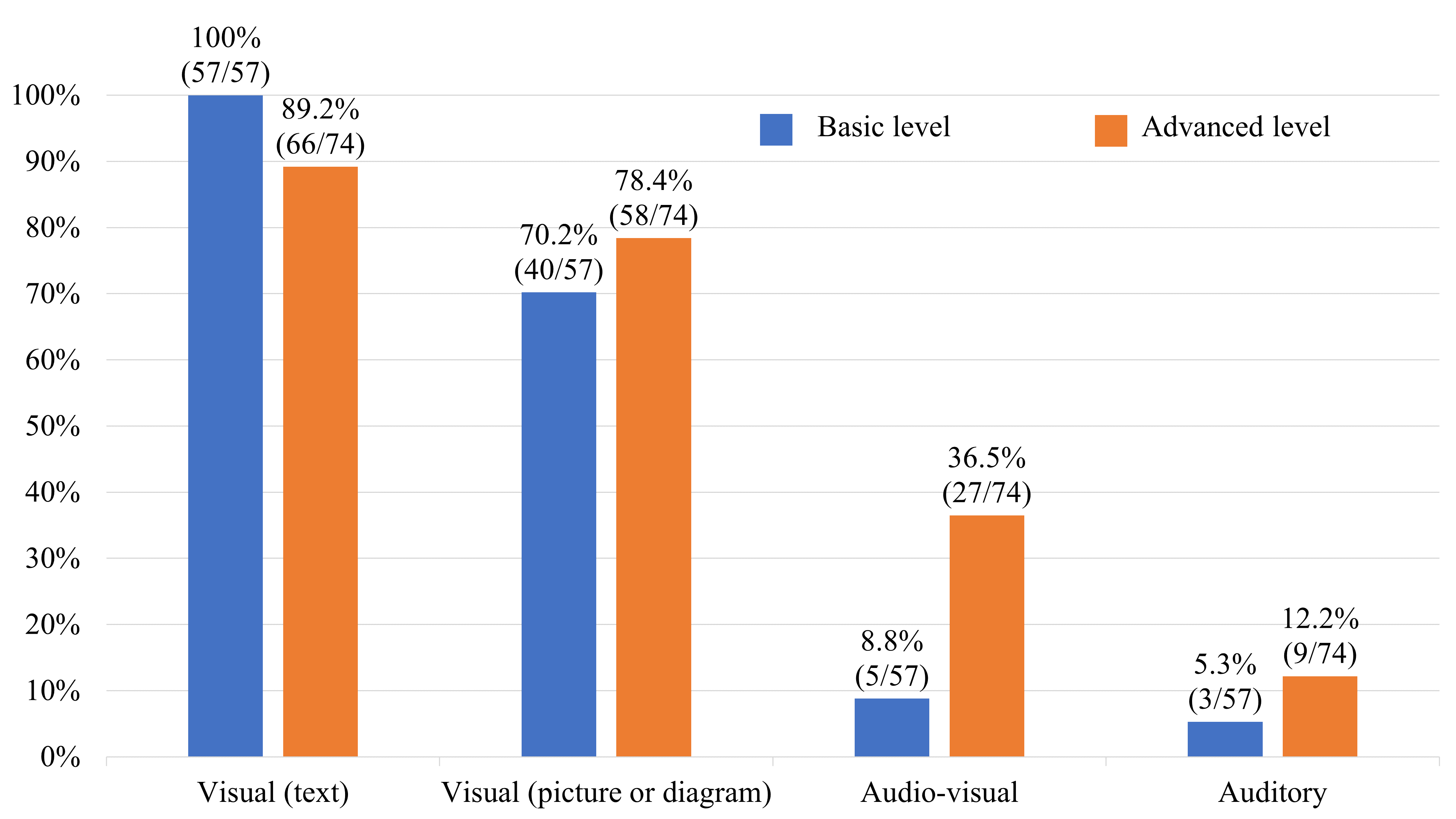


Comparison of learner levels according to learning styles; n=57 for basic level; n=74 for advanced level
